# A Flurry of Infectious Disease Modeling Tools During the COVID-19 Pandemic, Considerations for Future Selection

**DOI:** 10.3390/pathogens14090877

**Published:** 2025-09-02

**Authors:** Jennifer Corbin, Audrey Cerles, Peyton Tebon, Michael Haverkate, Susan Campbell, John Hurst, Rachel Woodul, John Hunzeker, Kierstyn Schwartz-Watjen, Akeisha Owens, Aiguo Wu

**Affiliations:** 1 Aeris, LLC, Louisville, CO 80027, USA; ptebon@aerisllc.com; 2 Gryphon Scientific, Takoma Park, MD 20912, USA; 3 Battelle Memorial Institute, Columbus, OH 43201, USA; woodul@batelle.org; 4 Applied Research Associates (ARA), Albuquerque, NM 87110, USA; jhunzeker@ara.com (J.H.); kierstynschwartz@gmail.com (K.S.-W.); 5 Defense Threat Reduction Agency (DTRA), Fort Belvoir, VA 22060, USA; akeisha.n.ownes.civ@mail.mil

**Keywords:** model selection, epidemiological models, infectious disease outbreaks, modeling tools

## Abstract

Infectious disease modeling surged during the COVID-19 pandemic, with numerous epidemiological models developed to shape both research and public policy. The abundance of available models—developed with diverse characteristics and modeling objectives—gave users plenty of model selection options; however, little guidance was available for selecting an appropriate epidemiological model. In recognition of this need for guidance, this work describes the development of a decision framework for appropriate model selection. To serve as both an example and a starting point for model users, we walk through the creation and use of a decision framework to evaluate key considerations for selecting a forward-looking epidemiological model intended to inform policy. Our assessment includes 43 models and modeling platforms that have been or could be used to model infectious disease. The framework developed for this assessment focused on assessing each model’s strengths and weaknesses in terms of flexibility and customization, the ability to implement pharmaceutical and non-pharmaceutical mitigations, and visualization capabilities. By providing a decision framework and demonstrating its use, we aim to support better decision-making and stronger trust between public health institutions and the constituents they serve.

## 1. Introduction

Infectious disease modeling dates back to the early 20th century, when the first SIR (Susceptible-Infected-Recovered) model was proposed [1]. Over the years, infectious disease modeling has become increasingly sophisticated, allowing researchers to create more realistic and data-driven models. The onset of the SARS-CoV-2 pandemic (COVID-19) in 2020 caused a surge in both the development and use of infectious disease models, driven by the desire to answer pressing questions from researchers and policymakers [2]. The models developed during this time were created by modelers across disciplines with different objectives, resulting in the emergence of a variety of epidemiological models, each with their own strengths, weaknesses, and specifications. While this variety advanced the field of epidemiological modeling through the creation of novel approaches, frameworks, and data ingestion pipelines, it also highlighted the importance of vetting model quality and using models appropriately. As researchers and policymakers turned to these newly developed models to understand the health risks posed by SARS-CoV-2, not all models were used appropriately with full consideration of their intended purpose or capabilities [2]. As a result, the use of modeling to inform highly consequential policies—such as travel restrictions, testing protocols, vaccine rollouts, and public closures—yielded mixed results [3].

Without guidance to indicate which modeling tool is most appropriate for an organization’s needs, model selection may be limited to factors such as access or institutional affiliation rather than the model’s intended use, capabilities, or appropriateness. During the SARS-CoV-2 pandemic, models developed by well-known institutions were often used when advising policy, even when, based on their intended use, they may not have been the most appropriate tool [4]. In some cases, this led to decision making informed by general models that failed to account for specific intricacies related to a state or region [5,6]. The reliance of policy makers on models that were not ideally suited to answer their questions produced conflicting results, resulting in decreased trust in public health organizations [4,7].

Previous efforts have sufficiently reviewed the technical aspects of modeling tools based on specific capabilities (i.e., data visualization) [8] or their structure (agent-based, compartmental, stochastic, etc.) [9,10,11,12] without addressing the broader range of characteristics that might influence model selection or providing guidance regarding the selection of a model [13]. Here, we provide a “how-to” framework to be used by researchers and policymakers when selecting epidemiological models for future outbreaks. We then implement the decision-making guidance to assess 43 existing models across key criteria that should be considered when selecting an infectious disease model. Using this framework and our initial assessment, we hope to enable researchers and policymakers to make informed and appropriate model selections based on the user’s needs and each model’s capabilities.

## 2. Methods

### 2.1. Development of the Decision Framework

The decision framework described for selecting an appropriate epidemiological model is adapted from the engineering design process [14]. The first steps of the process entail the identification of the specific problem at hand and the formation of criteria and constraints. Criteria included in Table 1 were based on the authors’ collective prior experience selecting, building, and using epidemiological models. A rational decision-making process is then suggested to evaluate models against the established criteria to minimize bias. The results of the evaluation should then be used to identify one or several models for further testing or use.

### 2.2. Identification of Models for Assessment

Infectious disease modeling tools were identified using a search of the academic literature and conference abstracts, reference mining of review papers, additional web searches, and a review of COVID-19 models catalogued by the Centers for Disease Control and Prevention’s (CDC) Center for Forecasting and Outbreak Analytics (CFA) [15] and Epidemic Prediction Initiative (EPI) [16].

Literature searches were conducted using the Google Scholar and PubMed databases with search terms such as “modeling,” “predictive,” or “forecast,” combined with terms like “epidemiology” or “disease.” To capture any models that were in use during the COVID-19 pandemic that were not identified via our literature review, we turned to the CFA and EPI catalogs of infectious disease models and additional web searches. Full texts were retrieved for those models that appeared to meet our inclusion criteria (see Supplementary Table S1). The references of the applicable literature were mined to find additional relevant studies.

We limited our assessment to models published in the academic literature, conference proceedings, or those described elsewhere between 2015 and 2023. This example was further constrained to include only those tools which modeled multiple agents, could be readily adapted to other agents, or allowed user-defined disease parameters; allowed for variation in spatial scale; could incorporate pharmaceutical and non-pharmaceutical interventions and behavioral modifications; or could support different outputs and visualizations. With the exception of EpiGrid [17], all models identified during the literature and publication review that met the initial criteria were included in the evaluation.

### 2.3. Evaluation of Models

The models included in our evaluation were assigned an ordinal ranking for each of the characteristics listed in Table 2. Models with more features or a more robust suite of choices scored high in the modeling flexibility categories, models that contained only some of the described functionality or offered reduced choices to the user were ranked as medium, and models that did not contain the described functionality or presented limited to no choice to the user were ranked as low. The visualization and mitigation criteria were similarly ranked, with greater functionality and user choice achieving a high ranking, some functionality or a reduction in user choice receiving a medium ranking, and lack of functionality receiving a low ranking.

## 3. Creation of a Decision Framework

In our framework, the first step in the selection of an appropriate epidemiological model is the creation of criteria specific to the use case. The criteria can include a wide array of factors, ranging from model accuracy under particular circumstances to the level of expertise required to operate the software. Table 1 illustrates example criteria that individuals might consider when developing a decision framework. It is neither exhaustive nor prescriptive. In practice, a small set of carefully chosen criteria is often sufficient to support a well-informed decision.

The process of selecting and prioritizing criteria is critical for identifying the best available options based on a user’s needs. While we believe an explicit ranking is often unnecessary, distinguishing between essential criteria and “nice-to-haves” can significantly streamline decision making. Clarifying which constraints are non-negotiable can help accelerate the process by establishing firm rule out conditions that quickly eliminate unsuitable options.

Once a decision framework has been established, specific models can be evaluated against the prioritized criteria. This begins with identifying clear metrics for each criterion, ideally using objective or quantifiable measures. Objective metrics—such as the presence or absence of features—can often be assessed with simple yes/no questions. Subjective measures, such as the level of expertise required for operation or interface usability may also be included, but care should be taken not to overweight these factors in the final decision-making process.

After evaluating all models against the pre-selected criteria and eliminating those that fail to meet key requirements, the user can choose one or more models that either excel in a specific area or demonstrate high performance across multiple high-priority criteria. When evaluating models, it is important to remember that each model should be evaluated according to the criteria and metrics for the defined use case and different models will perform better for different applications. If modeling objectives change, criteria must be reselected, and models must be reevaluated according to the newly established metrics to make the optimal choice.

Notably, our decision-making framework omits the assessment of model accuracy, precision, and efficacy to instead focus exclusively on the suitability of the model. Once users have identified an appropriate model for their specific circumstances, further technical assessment of the model is warranted to ensure the model is scientifically valid—this assessment is beyond the scope of this paper.

## 4. Demonstration of the Decision Framework for Selected Models

To demonstrate how a model selection framework can be used, we selected criteria for evaluating modeling tools that would be broadly applicable for forward-looking epidemiological modeling exercises intended to inform policy, such as those used during the COVID-19 pandemic. The EpiGrid model [17] has been widely used at the U.S. federal level for this purpose [18,19,20]; therefore, we sought to identify complementary solutions that could enhance current capabilities or address potential gaps. To this end, we selected three main categories upon which to base our assessment—flexibility, visualizations, and mitigations.

The flexibility category was selected because forward-looking, preemptive modeling tasks with large uncertainties about transmission dynamics require the versatility to handle a variety of emerging infectious diseases that may arise. Models capable of simulating multiple agents across a variety of geographic regions and time scales are well suited to these modeling tasks. The second category, visualizations, was selected because the creation of clear, interpretable figures that convey meaning to both technical and non-technical personnel is critical to the translation of research to policy. While there are several methods for developing visualizations—some integrated into the modeling software, others external—integrated tools offer ease of use, a reduction in troubleshooting, and they minimize the risk of misinterpretation. Finally, the inclusion of mitigations was selected as the third category for this example framework. Mitigations span a broad set of pharmaceutical and non-pharmaceutical countermeasures, and the impact of these interventions are of primary interest to policymakers taking action to reduce caseloads, minimize disease burden, and stop disease spread [21].

Though these three criteria were selected based on our specific scenario’s needs, other categories may be more useful under different circumstances. For example, researchers interested in intra-country spread may demand high geographic resolution models with subpopulations [22,23,24,25,26,27,28,29,30,31,32] while influenza vaccine developers may require disease-specific, long-term seasonal models for market size and revenue forecasts [27,29,31,33,34,35,36,37,38].

The models that were evaluated were identified through literature searches, reference mining reviews, or less formal search strategies. In our analysis, we included 43 models published in the academic literature, conference proceedings, or those described elsewhere between 2015 and 2023. Supplementary Table S1 outlines the basic information on each model (including the agents that can be modeled, the modeling type, and notes for the evaluation criteria). Given the needs of our modeling scenario, we only included tools capable of modeling multiple agents, allowing for variation in spatial scale, incorporating mitigations, and supporting visualization—lack of any of these essential characteristics were treated as automatic disqualifiers. Additional information on model selection is available in the methods section. Table 2 outlines the full set of model features and the specific evaluation metrics used to assess each model.

### 4.1. Flexibility

For the purposes of our evaluation framework, flexibility within any model is the ability to adapt to different needs specified by the individual user. Models were scored within each criterion, independent of other capabilities the model possessed. Models that scored highly in categories associated with flexibility represent those that have significant depth or breadth of use compared to others.

#### 4.1.1. Agent/Pathogen Selection

Flexibility in agent/pathogen selection allows a single model to be applied in a range of scenarios. Several of the models we evaluated have the capability to model any agent or pathogen, while others included only respiratory agents or vector-borne agents.

The evaluated models with the greatest flexibility for agent/pathogen selection, along with the agents they are capable of modeling, are listed in Table 3. Most models that excel in this category have the capacity to model any disease or agent. These models achieve maximum agent flexibility by allowing the user to specify the statistical parameters that the model uses to define a disease. While these models often have pre-programmed parameters for specific pathogens, user-derived specifications are useful in the application of modeling tools for previously uncharacterized diseases and strains. The ability to model both well-characterized and novel pathogens is particularly valuable in assessing tools used to prepare for future outbreaks.

#### 4.1.2. User-Defined Parameters

Models accept a range of user-specified data, with more focused models often including hardcoded disease parameters while more flexible models allow parameters to be modified by the user. Compartmental models and models with underlying compartmental frameworks often allow users to specify rates of movement between compartments, with rates for moving between susceptible, exposed, infected, and recovered compartments each adjusted separately. Models that require user-specified parameters require more research prior to modeling, whereas models that have parameters built into the model can be quickly implemented so long as the parameters have been sufficiently justified by the model developers (Supplementary Table S1). While predefined models require less input initially, they are only useful for the specific circumstances for which they were designed. In this sample evaluation, we highly rated models with the capability to modify and tune model parameters easily. Table 4 lists five of the models we evaluated that scored highly in the user-defined parameters category.

One additional consideration related to user-defined parameters is the availability and accessibility of source code. Open-source models are the most flexible, as users can not only change parameters, but also change functions and relationships that may be fixed in many other models. Though these models offer enormous potential for customization, they can require extensive subject matter expertise and coding experience to effectively manipulate.

#### 4.1.3. Geographic Capabilities

The infection rates and natural history of disease may vary according to geographical features or due to differences between populations living in specific regions. Factors such as rainfall, temperature, and proximity to livestock can all change the spread of disease between different populations. A model with the capabilities to account for these differences represents a useful tool when investigating disease spread across various geographical areas.

Models that can adapt to any spatial scale lend themselves to research questions that compare the impacts of large population disease spread on smaller communities within the population; although expanding geographical range typically reduces granularity and can increase both computational load and uncertainty. Likewise, modeling efforts that compare infection transmission and outbreaks in different populations defined by variations in area-level characteristics offer additional insights into the explanatory factors associated with disease transmission. Three of the models we evaluated that were particularly adept at being adapted to a wide range of geographic locations can be found in Table 5.

### 4.2. Visualization Capabilities

A model’s visualization capabilities are an integral component of data interpretation and allow the user to better understand and present the modeling results. Given the relative abundance of external visualization tools, the majority of models investigated produce data as numerical values in CSV or similar files. These computer-readable files are ideal inputs for secondary visualization software but may require additional expertise and time to develop into full visualizations. Furthermore, models that directly display data within their interface have the additional benefit of allowing users to understand their results within a controlled platform. Researchers that want to rapidly adjust parameters and visualize changes will likely find success with models that display data directly within their interface. In addition to simply having the ability to generate figures, the types of visualizations that the model can output should be considered when evaluating the model’s ability to convey results: geographical displays of data such as maps answer different questions than charts that align cases with resource availability. Of the models we analyzed, only the two models described in Table 6 demonstrated strong capabilities in generating useful visualizations.

### 4.3. Mitigations

When modeling the spread of infectious disease, it is important to consider both pharmaceutical and non-pharmaceutical interventions [44,45]. Pharmaceutical interventions, such as vaccines, antibiotics, and antivirals, can directly impact disease transmission and severity by modifying the disease of individuals [46]. Non-pharmaceutical interventions, such as social distancing, mask wearing, and hand hygiene, also play a role in slowing the spread of disease, exerting their effects by acting on disease transmission between individuals [21]. Incorporating these strategies into a model can provide more accurate and realistic projections of an outbreak’s progression and allow for more informed and accurate decision making. Several of the models we evaluated ranked highly when considering the implementation of pharmaceutical and non-pharmaceutical mitigations. Table 7 provides an overview of these tools.

## 5. Discussion

Modeling can be used for a variety of purposes, including driving policy decisions, assessing preventative measures and mitigations, forecasting logistical and supply needs, and estimating expected illnesses and deaths from an outbreak. As evidenced during the COVID-19 pandemic, shortcomings in the applications of models hindered public opinion on the reliability and usefulness of infectious disease modeling, leading to mistrust in public health organizations [4]. Given the gap in guidance on the fundamental problem of epidemiological model selection, we described how a structured, customizable framework can be implemented to select an appropriate model based on the research or decision-making objective. To illustrate the value of a structured framework for selecting an appropriate infectious disease model, we applied the framework to evaluate 43 models for their suitability in forward-looking epidemiological modeling. Each model’s strengths and limitations were assessed, and our results show that only a small subset of models emerged as top candidates for modeling the scenario we analyzed, helping to narrow the field for model selection. The full analysis is provided (Supplementary Table S1) to help researchers and end users build on this work—identifying additional requirements and constraints specific to their use case to select the most appropriate model.

The goal of this framework is not to offer a prescriptive solution for the selection of a single ideal model, but rather to push modelers to weigh contrasting needs and priorities and introduce objectivity when evaluating options. In our demonstrated use case, we identified several models that ranked highly across multiple categories, for example EMOD and STEM—and to a lesser extent, DICE, GLEAMViz, and IHME. While this approach is not intended to identify the single best model, it is useful in reducing the number of models under consideration, in this case narrowing model options from 43 to 5. By carefully constructing evaluation criteria and metrics, model users can avoid misapplying models—for instance by using incorrect time scales, incorrectly sized populations, or by attempting to model diseases beyond the model’s capabilities. While this paper focuses on the importance of using models only within their intended scope, it is important to recognize that even appropriately selected models can produce inaccurate results if the underlying parameters are not scientifically valid or are based on an incomplete understanding of a disease. While evaluation of a model’s accuracy is beyond the scope of this paper, prior to the implementation of a model, a systematic assessment of model performance should be conducted.

Even well-suited models may have limitations that prevent them from being effectively implemented. One major consideration is data availability and reliability, including both the distribution and quality of the data. Data constraints include the need for frequent updates to case counts, mobility patterns, and healthcare capacity, as well as limitations in standardized case definitions, timely data sharing, and the availability of granular data in resource-limited settings. The timeliness and reliability of this information is essential for accurate modeling but is often hindered by relying on surveillance data.

In cases where data is limited, it is imperative that uncertainty is both understood and explicitly conveyed with results. For policymakers, this uncertainty and can be identified by asking practical questions: How confident can we be in the projected case numbers? How likely is it that observed trends reflect real transmission dynamics versus artifacts of incomplete reporting? For example, during the COVID-19 pandemic, inconsistencies in surveillance and underreporting in some countries led to biased inputs, which in turn produced forecasts that underestimated transmission intensity and delayed recognition of surge risks.

Predicting future events is inherently uncertain and when input data are sparse or biased, ensuring that imprecision is clearly reflected in the outputs is crucial to translation to policy [48]. Forecasts may therefore need to present confidence intervals or scenario ranges rather than single point estimates which allow decision makers to prepare for a spectrum of possible outcomes rather than assuming one “correct” trajectory. Other strategies to address uncertainty include the concurrent use of multiple models (or sets of modeling parameters) to generate ensembles to get a better understanding of likely trends versus model artifacts. During COVID-19, ensemble approaches produced more accurate forecasts of deaths than any individual model included in the ensemble [49]. The integration of multiple modeling strategies is beneficial because no single model can fully capture the complexity of an emerging outbreak, especially when data are sparse or evolving. Individual models may be overly sensitive to specific assumptions, such as transmission rates, intervention effectiveness, or reporting delays. By combining outputs across diverse models, ensembles tend to “average out” extreme predictions, reduce the influence of model-specific biases, and provide more stable and reliable forecasts. By utilizing ensembles or framing uncertainty in terms of risks and ranges, rather than exact numbers, officials are better positioned to weigh policy options under imperfect information.

## 6. Conclusions

As we collectively move forward from the COVID-19 pandemic and prepare for future infectious disease outbreaks, it is important to re-evaluate public health responses and improve them where necessary. The use of epidemiological models to forecast caseloads, convey risk, and shape policy is a highly consequential area where public health institutions can improve their practices and communication to increase trust with their constituents. To facilitate the process of identifying appropriate models and selecting the best tool for the desired goals, we outline a structured framework for model selection and advocate for subsequent technical evaluation of model options to ensure the tool has both the necessary capabilities and also aids users in translating results into action. Using the framework outlined above, researchers and policymakers can make better-informed decisions about epidemiological modeling to support planning and response initiatives.

## Figures and Tables

**Table 1:** List of criteria for consideration when selecting an epidemiological model.

Category	Possible Criteria
Epidemiological Factors	- Pathogens included - Population characteristics ○ Subpopulation inclusion ○ Prior/waning immunity ○ Non-human hosts - Geographic/environmental compatibility - Temporal suitability ○ Long range vs. short range - Temporal resolution (hours, days, weeks, etc.)
Inputs and Outputs	- Inputs ○ Outbreak case data ○ Transmission parameters - Outputs ○ Metrics and level of detail ○ Frequency ○ Uncertainty
Model Structure	- Model type (compartment, agent-based, statistical) - Stochastic or deterministic
Computational Requirements	- Computational expense - Software required - Ability to update/Open source
User Interaction	- User interface - Level of expertise required - Model run time
Flexibility	- Can model multiple agents - Modifiable transmission and disease parameters - Modifiable populations - Geographic/temporal scalability
Mitigation	- Medical and non-medical countermeasures - Timing of interventions - Behavioral changes - Partial interventions and missed doses
Visualization	- Visualization tools - Interactive dashboards - Standardized output formats - Automated report generation
Credibility	- Accuracy ○ For a region of interest ○ For time period of interest ○ For specific circumstances - Data sources and quality - Assumptions - Reproducibility - Developers - Documentation - Public perception

**Table 2:** Evaluation criteria for example assessment.

Category	Subcategory	Model Feature	
Flexibility	Has the ability to adjust or model:	A generic respiratory disease	
		A novel disease	
		Diseases that would likely follow a natural disaster	
		Vector-borne diseases	
	Has the ability to adjust or model the following disease parameters:	Disease-specific parameters (e.g., R_0_, incubation period)	
		The effect of waning immunity, as seen in COVID-19	
		The impact of multi-strain or change of (dominant) strain of agents	
		Co-infection with two pathogen variants	
		For vector-borne diseases, the variation of vectors under climate change scenarios	
		For vector-borne diseases, growth of the human population or changing interactions with the vector species	
	Has the ability to adjust or model the following transmission parameters:	Disease seasonality	
		Whether susceptibility or mortality is higher as a result of co-infection	
		Age differences in susceptibility	
		Sex differences in susceptibility	
		Modification of susceptibility in sub-populations (e.g., stratified by age/sex) and over time	
		Contributions of vectors and animal reservoirs to disease transmission	
		Major modes of transportation in the area	
		Forecast disease incidence based on seasonal trends	
	Has the ability to adjust or model the following population parameters:	Disease spread within a small population (<5000 people)	
		Customizable population size	
		Behavioral parameters of the population (e.g., patterns of social interaction)	
Visualizations	Has the following visual elements:	Aspects/attributes of the model provide visualization for forecasts	
		Alternative methods for presenting an epidemiological outbreak, besides traditional epidemic curves	
Mitigations	Has the following mitigations:	The ability to adjust or model pharmaceutical interventions	
		The ability to adjust or model non-pharmaceutical interventions	
		Impact of medical response on the outbreak progression can be modeled	
		Impact of foreign assistance withdrawal on the spread of disease can be modeled	

**Table 3:** Models with high flexibility scores in agent/pathogen selection.

Model Name	Model Developer	Agent List
Dynamics of Interacting Community Epidemics (DICE) [24]	Predictive Science Inc., San Diego, CA, USA	Chikungunya; Cholera; COVID-19; dengue; Ebola; Influenza; Lassa fever; Lyme disease; measles; MERS-COV; plague; SARS-CoV; yellow fever; Zika
Epidemiological MODeling (EMOD) [26]	Institute for Disease Modeling, Bill and Melinda Gates Foundation, Seattle, WA, USA	Any
EpiNow2 [39]	London School of Hygiene and Tropical Medicine London, United Kingdom	Many, no native support for VBDs
Epistemix (FRED) [27]	University of Pittsburgh Pittsburgh, PA, USA; Epistemix, Pittsburgh, PA, USA	Any
GLEAMViz [34]	Northeastern University Boston, MA, USA	Any
Kendrick [40]	KendrickOrg, Atlanta, GA, USA	Any
Lightweight Infectious Disease Simulator (LIDS) [41]	University of Thessaly Volos, Greece	Any

**Table 4:** Models with many user-defined parameters.

Model Name	Model Developer	User-Defined Parameters
CommunityFlu 2.0 [33]	CDC Atlanta, GA, USA	Users can modify any of 320 inputs. Accounts for age (children, adults, and the elderly).
Eclipse Spatio-Temporal Epidemiological Modeler (STEM) [25]	IBM Corporation, Armonk, NY, USA; Eclipse Foundation, Brussels, Belgium	Users can input agent-specific parameters (e.g., transmission rates, mortality rate, loss of immunity).
Epidemiological MODeling (EMOD) [26]	Institute for Disease Modeling Bill and Melinda Gates Foundation, Seattle, WA, USA	The user can define populations and their demographics/geographies. The user can define aspects of the simulation, including disease characteristics like infectivity and route of transmission. Each agent (such as a human or vector) can be assigned a variety of “properties” (for example, age, gender, etc.).
PatchSim [42]	University of Virginia Charlottesville, VA, USA	Users can control parameters across space and time with week-to-week and month-to-month granularity.
PyPM [36]	University of Victoria Victoria, BC, Canada; TRIUMF, Vancouver, BC, Canada	Most parameters can be input by users. Compartments can be modified to users’ specifications.

**Table 5:** Models with broad geographic capabilities.

Model Name	Model Developer	Geographies	Spatial Granularity
Eclipse Spatio-Temporal Epidemiological Modeler (STEM) [25]	IBM Corporation Armonk, NY USA; Eclipse Foundation, Brussels, Belgium	Any	Country; state; county
Epidemiological MODeling (EMOD) [26]	Institute for Disease Modeling Bill and Melinda Gates Foundation Seattle, WA, USA	Any	User-defined for any scale from hyperlocal (e.g., household) to regional to national
SIkJalpha [30]	University of Southern California Los Angeles, CA, USA	Any (country); US (county, state)	Any (e.g., hospital, city, county, state, country)

**Table 6:** Models with strong visualization capabilities.

Model Name	Model Developer	Visualizations
GLEAMViz [34]	Northeastern University Boston, MA, USA	Output with maps, charts, and other useful graphics.
IHME COVID-19 Model [43]	Institute for Health Metrics and Evaluation Seattle, WA, USA	Curves for each output for all selected locations, maps for locations within a region with locations color-coded according to outputs.

**Table 7:** Models that allow for significant modeling of pharmaceutical and non-pharmaceutical mitigations.

Model Name	Model Developer	Mitigations and Interventions
Eclipse Spatio-Temporal Epidemiological Modeler (STEM) [25]	IBM Corporation Armonk, NY USA; Eclipse Foundation Brussels, Belgium	Able to incorporate diverse datasets, including real-time data (e.g., weather and environmental data). Can model spread across years for pandemics. Includes examples of model parameterization for notable diseases of interest, including endemic, emerging infectious, and vector-borne diseases.
Epidemiological MODeling (EMOD) [26]	Institute for Disease Modeling, Bill and Melinda Gates Foundation Seattle, WA, USA	The user can define the interventions that take place. Complex interventions can be modeled (e.g., cascade of care for HIV infections). Mitigations can be applied to specific subpopulations.
IHME COVID-19 Model [43]	Institute for Health Metrics and Evaluation Seattle, WA, USA	Shows how policy decisions impact the trajectory of COVID-19. Factors in important drivers of trends in COVID-19, such as vaccination rates, mobility data, and self-reported mask use. Accounts for vaccine usage and vaccine efficacy against different variants. Can model the efficacy of antivirals.
Imperial College London COVID-19 Model/Epidemia [47]	Imperial College London London, United Kingdom	Can model the effect of non-pharmaceutical interventions, especially focusing on mobility and social interaction, and assumes that changes in Rt are an immediate response to interventions. Can also model the effect of pharmaceutical interventions.
Local Epidemic Modeling for Management and Action (LEMMA) [29]	UC Berkeley Collaboration Berkeley, CA, USA	Supports long-term projections. Users may specify the timing and impact of various public health interventions, such as school closures and shelter-in-place orders. Interventions may occur before the current date to reflect public health interventions. Interventions may also occur after the current date and can be used to simulate the future course of the epidemic if measures are implemented or lifted at a future date. Multiple interventions may be applied at the same time. Vaccination data may be incorporated.

## Data Availability

No new data were created or analyzed in this study. Data sharing is not applicable to this article.

## Supplementary Materials

The following supporting information can be downloaded at https://www.mdpi.com/article/10.3390/pathogens14090877/s1, Supplementary Table S1: Models included in study.

## Abbreviations

The following abbreviations are used in this manuscript:

CDC	Centers for Disease Control and Prevention
CFA	Center for Forecasting and Outbreak Analytics
COVID-19	SARS-CoV-2 pandemic
DICE	Dynamics of Interacting Community Epidemics
EMOD	Epidemiological MODeling
EPI	Epidemic Prediction Initiative
FRED	Framework for Reconstructing Epidemic Dynamics – Model owned by Epistemix
LEMMA	Local Epidemic Modeling for Management and Action
LIDS	Lightweight Infectious Disease Simulator
SIR	Susceptible-Infected-Recovered
STEM	Eclipse Spatio-Temporal Epidemiological Modeler
